# Comparing Thriving at Work Among Trans-Tasman Early-Career Nurses: A Multinational Cross-Sectional Study

**DOI:** 10.3390/healthcare14030313

**Published:** 2026-01-27

**Authors:** Willoughby Moloney, Daniel Terry, Stephen Cavanagh, Stephen Jacobs

**Affiliations:** 1 Betty Irene Moore School of Nursing, University of California Davis, Sacramento, CA 95817, USA; sjcavanagh@health.ucdavis.edu; 2 School of Nursing and Midwifery, University of Southern Queensland, Toowoomba 4350, Australia; daniel.terry@unisq.edu.au; 3 Centre for Health Research, University of Southern Queensland, Toowoomba 4350, Australia; 4 Institute of Health and Wellbeing, Federation University Australia, Ballarat 3353, Australia; 5 Medical and Health Sciences, School of Nursing, University of Auckland, Auckland 1030, New Zealand; s.jacobs@auckland.ac.nz

**Keywords:** thriving at work, burnout, retention, early-career nurses, nurses, authenticity at work, quality of care, colleague support, workforce development

## Abstract

**Highlights:**

**What are the main findings?**

- Early-career nurses in New Zealand experience higher levels of burnout and report colleague support as the key predictor of thriving at work, highlighting the need for workplaces to reduce workloads and foster social connection.
- In Australia, authenticity at work is the key predictor of thriving, highlighting the importance of organisational cultures that enable nurses to express their true selves and professional identity.

**What are the implications of the main findings?**

- Applying the Thriving at Work model to the New Zealand and Australian nursing context highlights important differences that organisations should address when developing workforce policies in each country.
- The issue of early-career nurse attrition from the profession is an international concern. This research provides an innovative and theory-informed approach to examining how organisations can better support early-career nurses, which can be used by health policy makers internationally.

**Abstract:**

**Background/Objectives**: The Thriving at Work model proposes that organisations have a responsibility to provide supportive work environments that identify individual health outcomes, which organisations can use to determine where workforce support is needed. The aims of this study are to (1) identify and compare the predictors of early-career nurses’ thriving at work in New Zealand and Australia and (2) provide innovative and theory-informed recommendations to improve organisational support of early-career nurses to increase retention in the profession. **Design:** A multinational cross-sectional study design was followed. **Methods**: The methods include a sub-study of an international action research programme to support the thriving of early-career nurses, which evaluates and compares results from surveys of nurses at approximately three months post-registration in 2024 and 2025. A theory-informed survey assesses predictors and outcomes of thriving at work. **Results**: Early-career nurses (*N* = 320) from New Zealand (*n* = 277) and Australia (*n* = 43) completed the survey. New Zealand early-career nurses experience greater quality of care and authenticity at work; however, they also report greater burnout. For Australian early-career nurses, authenticity at work is the greatest predictor of thriving. In New Zealand, thriving is linked to burnout and colleague support. **Conclusions**: New Zealand must focus on reducing burnout and fostering workplaces that value social connection if it wants to mitigate early-career nurse attrition to Australia for better working conditions. In Australia, the value of authenticity at work highlights the importance of organisational cultures that enable nurses to express their true selves and professional identity. The findings highlight the need for tailored approaches in each country to strengthen workforce sustainability and improve nurse wellbeing. **Implications for the Profession**: In New Zealand, additional funding to bolster the recruitment and retention of the nursing workforce is crucial to improve patient ratios and reduce workloads. The remuneration of nurses must also remain competitive with Australia. Additionally, workplaces should incorporate Māori values and practices into workplace policies to strengthen social connections. Australian organisations should include authentic management training, psychological safety initiatives, and policies that value diversity and encourage open communication.

## 1. Introduction

Nurses play a vital role in healthcare systems worldwide, and with continued growth in workforce numbers, are the largest occupational group within the health sector [1]. Despite this, a global shortage of nurses is projected to reach 13 million by 2030, driven by ageing populations, an ageing nursing workforce nearing retirement, increasing patient acuity and comorbidities, and high workforce attrition rates [2,3]. In New Zealand, hospitals are short an average of 587 nurses every shift [3]. Early-career nurses (ECNs), those within their first five years of practice, are particularly difficult to retain [4,5]. In the US, more than 22% of nurses leave within the first year after graduating [6] with similar levels of attrition in New Zealand and Australia [7]. Key reasons for voluntary resignations include stress, burnout, workload, working conditions, salary, and toxic leadership [3,6,8]. In New Zealand, less than half of new graduates are matched to supported-entry roles, and many depart for Australia for better pay and working conditions [3]. However, in Australia, these same workforce pressures persist, with voluntary resignations driven by stress, burnout, excessive workload, poor working conditions, inadequate remuneration, and negative leadership cultures [9].

Younger workers are particularly at risk of burnout due to distinct challenges as they establish themselves in their careers and deal with the complexities of balancing their personal and professional lives [10]. The current cohort of ECNs is also generally of the ‘Gen Z’ generation, who are typically more comfortable with setting clear work boundaries and are less likely to overwork or commit to organisations that do not care about their wellbeing [11]. This means leaders may have to adapt their management styles to retain younger workers [12]. In addition to its adverse effects on individual nurses, burnout is linked to diminished quality of patient care, decreased patient satisfaction, and reduced organisational commitment and productivity [13,14]. Approaches to dealing with the issue of burnout in nurses have typically focused on improving the resilience of individuals; however, evidence suggests that the focus should be on what organisations can do to improve working conditions for ECNs [15,16,17,18].

## 2. Background

This study is part of a larger international action research programme on thriving at work in nurses, which is guided by a shared theoretical framework [19]. The environment in which nurses work is complex, and novel solutions are required to engage with ECNs to investigate their views and include them in the development of interventions that reduce burnout and improve retention. The Institute for Healthcare Improvement (IHI) Framework for Improving Joy in Work [12] and the Thriving at Work model [20] provide the theoretical framework for this research. The Joy in Work framework proposes that work satisfaction is impacted by organisations. It promotes wellbeing by focusing on creating a joyful and engaged workforce through reducing burnout and other adverse factors in the work environment. By prioritising nurses’ concerns and co-developing strategies to improve the work environment, this four-step framework is well suited to the needs of ECNs. It is utilised in this study to (1) ask ECNs “what matters to you?”; (2) identify unique impediments to joy in work in the local context; (3) commit to a systems approach to making joy in work a shared responsibility at all levels of the organisation; and (4) use improvement science to test approaches that seek to improve joy in work in the organisation [21].

To achieve a systematic approach to ensuring organisational responsibility, the Socially Embedded Thriving at Work model [20] provides a theoretical systems approach. It identifies the personal and organisational factors related to thriving at work, working towards a reduction in turnover [22,23]. Thriving is achieved when workers experience a sense of (1) vitality, feeling energised and enthusiastic for work, and (2) learning and growing through acquiring new knowledge and skills [20]. The Thriving at Work model proposes that organisations should identify individual health outcomes to determine where workforce support is needed. This suggests that both organisations and ECNs themselves are responsible for their ability to thrive [24], and the resources available to them within organisations are a determining factor [25].

**Figure 1:**
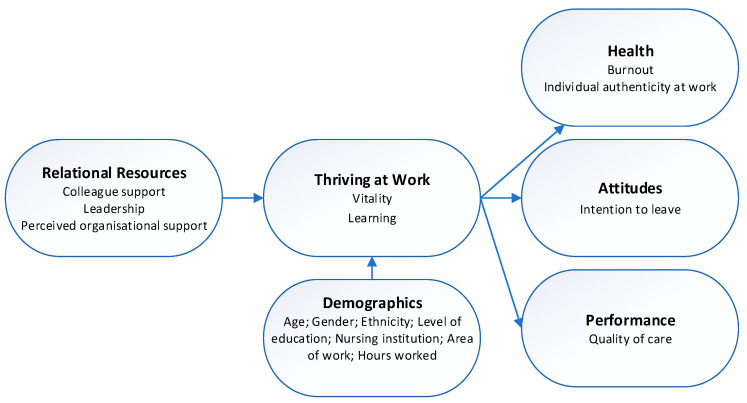
Adapted conceptual network of workplace antecedents and outcomes of thriving at work.

A meta-analysis of the Thriving at Work model identified a network of antecedents and outcomes associated with the model [22]. Antecedents of thriving at work are separated into individual characteristics and relational resources, while outcomes include health, attitude, and performance. An adapted model of the conceptual network of assumed antecedents and outcomes of thriving at work (Figure 1) informed the development of the standardised e-surveys in this study.

Given the evidence suggests that the focus should be on what organisations can do to improve working conditions for ECNs, this study focuses on the relational, or environmental, antecedents of thriving. These include colleague support, leadership, and perceived organisational support. Colleague support is characterised as actions by coworkers that provide instrumental benefits, help individuals cope with adversity, and support personal growth and development [22]. Colleague support fosters nurses’ thriving at work by enhancing both learning through supportive peer relationships that promote psychological safety and a sense of belonging, enabling them to express concerns, ask questions, and engage in reflective practice without fear of judgment [26]. Likewise, leadership plays a pivotal role in enhancing employees’ sense of thriving at work by fostering supportive, trusting, and development-oriented environments [22]. Supportive leadership is characterised by empowering staff and cultivating positive leader–staff relationships, resulting in reduced burnout and enhanced thriving, job satisfaction, organisational commitment, and retention [27,28]. Perceived organisational support describes employees’ beliefs regarding the extent to which their organisation values their contributions and cares about their wellbeing [29]. This perception of support encourages employees to invest more in their roles, develop new skills, and contribute beyond formal responsibilities, thereby promoting workplace thriving [22].

Improving nurses’ ability to thrive at work is linked to them feeling authentic at work, which may enable them to be more able to deal with job demands, such as high workloads that can threaten engagement with work and lead to cynicism, exhaustion, and burnout [30,31]. However, intention to leave is not always a sign of a negative work environment. If nurses are thriving, they may still have an intention to leave the organisation as they seek to gain new skills and experiences in different areas of nursing. Job-related factors such as work environment and support are associated with professional turnover intention, suggesting that nurses do not believe their sense of thriving will improve in another nursing role [32]. This study focuses on the attitudinal outcome of intention to leave the profession, where the skills and experience of nurses are lost to the profession. The performance outcome is quality of care because a higher degree of burnout has been shown to correlate with a reduced perceived patient care quality [33].

## 3. Study Aims

1. To identify and compare the predictors of early-career nurses’ thriving at work in New Zealand and Australia;
2. To provide innovative and theory-informed recommendations to improve organisational support for early-career nurses to increase retention in the profession.

## 4. Materials and Methods

### 4.1. Design

**Figure 2:**
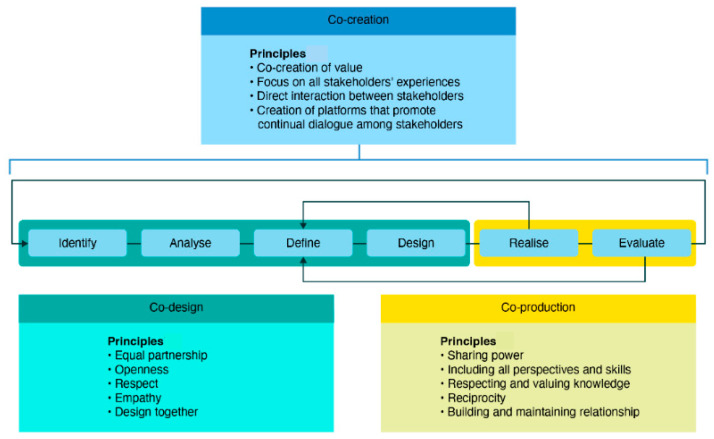
Co-creation model [35].

This was a multinational cross-sectional study. It sits within a broader international theory-informed participatory action research approach that integrates co-creation to support stakeholder-led change [34]. Co-creation meetings enable direct contact between stakeholders in which they follow strategic co-design and co-production guidelines (Figure 2) [35]. This actively engages ECNs and nurse leaders in identifying challenges, designing contextually relevant solutions, and evaluating progress through an iterative and collaborative process. By centring lived experience and fostering mutual accountability, the methodology aims to promote trust, shared ownership, and a stronger sense of belonging, factors anticipated to enhance ECN retention and professional growth [35].

The long-term research programme has three phases: (1) survey of ECNs to gather baseline data about key antecedents and outcomes of thriving at work, (2) focus groups with ECNs to explore the results of the survey in greater depth, and (3) co-design meetings between ECNs and nurse leaders to develop practical and sustainable solutions to the issues identified in the preceding phases. The cycle of data collection is repeated each year as new nursing cohorts begin working and up to five years of their employment. This paper reports the findings of the initial survey phase from the 2024 and 2025 ECN cohorts in New Zealand and Australia.

### 4.2. Study Setting and Sampling

In New Zealand, invited participants included all ECNs working at two health services in Auckland, New Zealand. The inclusion criteria were being a registered nurse who began clinical practice in the 2024 and 2025 intake, currently working clinically in the service jurisdiction, and willing to complete the survey in English. Similarly, all ECNs in Australia working at two health services in Southeast Queensland and one health service in Victoria, who commenced practice in the 2025 intake, were also invited to participate.

### 4.3. Data Collection

After approximately three months of practice, ECNs were invited to complete the comprehensive e-survey, informed by a meta-analysis of thriving at work [22] and containing validated tools to measure relational resources, health, attitude, and performance (Appendix A). Data were collected from ECNs in New Zealand (*n* = 172) and Australia (*n* = 43). In New Zealand, recruitment was conducted via email invitations sent by the research team in early 2024 using contact information provided by the organisations’ Nursing Workforce Learning and Development units. The email invitations included a link to a Qualtrics^XM^ survey, enabling ECNs to complete the questionnaire remotely and anonymously at their convenience. Informed consent was obtained electronically before survey completion. One reminder email was sent after two weeks. A similar approach was undertaken in Australia, where recruitment was via face-to-face presentations with ECNs undertaking education training days and via email invitation after approximately three months of practice. The same survey was administered to Australian participants, however, using the RedCap 16.0.0 survey.

#### 4.3.1. Thriving at Work

The 10-item Thriving at Work Scale [36] (reliability α = 0.88–0.94) measures the psychological state of thriving, encompassing feeling alive and energised (vitality) and a drive towards improvement (learning). It contains a 7-point scale (1 = strongly disagree, 7 = strongly agree), and when the 10 items are summed, it provides a total thrive score, which ranges between 10 and 70, where the higher the score, the greater the capacity to thrive.

#### 4.3.2. Burnout

The Malach–Pines Burnout Scale—short version (BMS-10) [37] (reliability α = 0.85) is a 10-item self-report instrument designed to assess burnout levels in individuals across various professions and settings. Each item evaluates the frequency of experiences related to physical, emotional, and mental exhaustion. A 5-point Likert scale ranging from 0 (never) to 4 (always) is used, with higher scores indicating greater levels of burnout.

#### 4.3.3. Colleague Support

The Colleague Support scale [38] (reliability α = 0.74) is a 4-item scale that consists of self-reported perceptions of the extent to which colleagues appreciate the nurse’s work, express positive evaluations, provide supportive advice, and assist with work-related tasks. It contains a 6-point Likert-type scale (1 = not at all, 6 = very much; 1 = never, 6 = very often), which provides a total score (4–24), with higher scores indicating greater perceived colleague support.

#### 4.3.4. Leadership

The Lead-Member Exchange (LMX-7) scale [39] (reliability α = 0.80 to 0.90) is a 7-item scale that measures the quality of a leader or manager’s working relationship with a worker. It is a self-reported scale of trust, mutual respect, and obligation to managers. It contains a 5-point Likert-type scale (1 = rarely, 5 = very often; 1 = strongly disagree, 5 = strongly agree), which provides a total quality relationship score ranging between 7 and 35, where scores of 30–35 are very high and 7–14 are very low.

#### 4.3.5. Organisational Support

The Survey of Perceived Organisational Support (SPOS) [29] (reliability α = 0.93) evaluates employee perceptions regarding their organisation’s support. The three items from the SPOS scale that loaded highest (factor loadings of 0.93) in the factor analysis by Wayne et al. [40] were used. It contains a 7-point scale (1 = strongly disagree, 7 = strongly agree), and when the three items are summed, it provides a total perception of organisational support score, which ranges between 3 and 23. The higher the score, the stronger the perceptions of organisational support among participants.

#### 4.3.6. Quality of Care

The Quality of Care scale [33] (reliability α = 0.71) is a 5-item (shortened) scale that assesses self-reported patient care measures, including frequency of errors and challenges related to time constraints. It contains a 5-point Likert-type scale (1 = never, 2 = once, 3 = a couple of times, 4 = multiple times and 5 = often), and when the five items are summed, it provides a total quality of care score, which ranges between 5 and 25.

#### 4.3.7. Individual Authenticity at Work

The Individual Authenticity at Work scale [41] (reliability α = 0.80) is a 6-item scale used to assess the extent to which individuals feel they can be their true selves at work. It contains a 7-point Likert scale ranging from 1 (strongly disagree) to 7 (strongly agree), producing a total score between 6 and 42, with higher scores indicating greater authenticity.

#### 4.3.8. Intention to Leave the Profession

Three items from the Intention to Leave scale [42] evaluate nurses’ behavioural short-term and long-term intentions to leave the nursing profession (reliability α = 0.78). Each item is rated on a 7-point Likert scale ranging from 1 (strongly disagree) to 7 (strongly agree). The summed scores produce a total intention-to-leave score, with higher scores indicating a stronger intention to leave nursing.

#### 4.3.9. Demographics

Demographic information was gathered, including birth year, gender, race/ethnicity, highest level of education, and email for future contact.

### 4.4. Data Analysis

Quantitative data were downloaded from Qualtrics and RedCap as Excel files. The data were then cleaned, checked, and analysed using Statistical Package for the Social Sciences (SPSS, Version 25.0). Descriptive statistics were performed, along with an independent samples *t*-test to compare New Zealand and Australia ECNs across variables. Further, multiple linear regression with backward elimination was also performed, starting with all variables and the least significant variables being removed individually until no further improvement was observed [43,44]. Analysis was undertaken to check for violation of the assumptions of normality or homoscedasticity; linearity was present where the scatterplot of standardised residuals met the assumptions, while multicollinearity was indicated by correlation coefficients greater than 0.8 and variance inflation factors less than 10 [45]. Overall significance was determined at a two-tailed *p* ≤ 0.05. A framework analysis approach [46] was applied to the qualitative free text data, conducting content analysis and aggregating data with the research question themes. Credibility was enhanced through regular peer debriefing among the research team.

### 4.5. Ethical Considerations

Ethics committee approval was obtained from the University of Auckland Human Participants Ethics Committee on 9 August 2024 (AH27200) and Darling Downs Health and Hospital (DDHH) Human Research Ethics Committee on 24 May 2024 (LNR/2024/QTDD/107282). Study participation was voluntary, and consent was assumed when the participant chose to complete the survey online. Data were stored and analysed anonymously.

## 5. Results

Demographic results are presented in Table 1. In New Zealand, a total of 277 ECNs completed the survey. The mean age of the participants was 27.6 years. The majority ethnicity was European/Pākehā, then Asian, Māori, and Pacific Peoples. Further, the majority worked in surgical medicine, then child health, general medicine, and mental health. In contrast, 43 Australian ECNs participated. Their mean age of 32.34 years was significantly higher than that of New Zealand ECNs. Their ethnicities were also significantly different, with a greater proportion of European background. Lastly, their clinical areas worked was significantly different, with a greater proportion of Australian ECNs working in general medicine. In addition, there was also a significant difference between the countries in terms of gender, with the highest proportion of females in Australia.

An independent sample *t*-test was conducted to compare New Zealand and Australia ECNs across variables (Table 2). Burnout was significantly lower among Australian ECNs compared to New Zealand ECNs. In contrast, authenticity at work was significantly higher among New Zealand ECNs. Similarly, quality of care was significantly higher among New Zealand ECNs compared to Australian ECNs. No other significant differences were found among any other factors, suggesting each of the factors were relatively similar to each other across both countries.

A multiple regression analysis was conducted to examine factors predicting thriving among ECNs in New Zealand and Australia (Table 3). For New Zealand, the model explained 61.2% of the variance in thriving (Adjusted R^2^ = 0.357). The significant predictors were burnout and colleague support. For Australia, the model explained 79.7% of the variance (Adjusted R^2^ = 0.572), with authenticity at work emerging as the only significant predictor.

In addition, when examining predictors of thriving among ECNs in New Zealand between cultural groups, distinct patterns emerged. For European (Pākehā) ECNs, burnout was a significant negative predictor of thriving, indicating that higher levels of burnout were associated with lower levels of thriving. Among Māori ECNs, colleague support was a significant positive predictor of thriving, which was also similar among Pacific peoples ECNs, suggesting that reduced perceived support from colleagues corresponded with diminished thriving among these two groups. In contrast, for Asian nurses, no variables emerged as significant predictors of thriving, and differences between cultures were not examined due to smaller numbers.

## 6. Discussion

### 6.1. Predictors of Thriving at Work

#### 6.1.1. Burnout

The results show that ECNs in New Zealand and Australia experience similar levels of thriving, support from colleagues, leaders and organisations, and intention to leave the profession. New Zealand ECNs report higher levels of quality of care and authenticity at work; however, they also experience higher levels of burnout than their Australian counterparts. While both New Zealand and Australia offer high standards of healthcare, the healthcare systems in each country have their own unique challenges. A major issue for the New Zealand nursing workforce is lower nurse-to-patient ratios than in other OECD countries. Adequate staffing ratios are essential for managing the complex and demanding workload of nurses, and research has consistently shown that higher nurse-to-patient ratios are associated with lower workloads per nurse, which results in less stressful working conditions [47]. Currently, New Zealand recommends a ratio of 1:4; however, it has not established legal minimum ratios, and nurses report that shifts across the country are understaffed most of the time [47]. In contrast, New Zealand nurses working in Australia report that mandated nurse-to-patient ratios make the job more satisfying [47]. Legislated nurse-to-patient ratios of 1:4 occur in Queensland and Victoria, with NSW, ACT, and South Australia soon to follow. Further to this, the New South Wales government is preparing to roll out a major staffing reform to boost the number of frontline healthcare workers in public hospitals, including a recruitment drive aiming to employ more nurses by 2027 under the state’s staffing levels major reform project [48]. In moves that are likely to compound burnout, in 2026, the New Zealand government will reduce clinical load-sharing hours, where ECNs work alongside senior nurses before taking on a full patient load and cut on-the-job training hours [49].

Taken together, these findings highlight burnout not only as an individual wellbeing issue but as a critical threat to nursing workforce sustainability. Elevated burnout among New Zealand ECNs, despite high reported quality of care and authenticity, suggests that professional commitment alone is insufficient to sustain them in environments characterised by high workloads and limited structural support. Persistent exposure to understaffing, reduced transition support, and constrained development opportunities risks normalising burnout early in nurses’ careers, which has long-term implications for retention, career longevity, and the capacity of the health system to maintain a stable and experienced workforce. Addressing burnout at the early career stage is therefore essential not only for protecting individual wellbeing but also for safeguarding the future supply and resilience of the nursing workforce.

#### 6.1.2. Colleague Support

Colleague support emerged as a significant predictor of thriving in New Zealand ECNs. This is in line with international research that shows social support from colleagues helps to mitigate stress and enhance the perception of organisational support [22,50]. Further, research on ECNs in New Zealand and Australia has concluded that positive and supportive relationships with colleagues improve thriving at work, and organisations should focus on fostering strong collegial connections to promote workplace wellbeing [51,52]. The higher importance of colleague support reported by New Zealand ECNs likely reflects the higher numbers of indigenous Māori and Pacific peoples in the New Zealand sample. Indeed, results show that colleague support is a significant predictor of thriving for Māori and Pacific peoples. This is unsurprising given the value placed on the concept of whanaungatanga amongst Māori, where forming connections and strengthening relationships to enhance a sense of belonging and togetherness is crucial to wellbeing [53].

The strong association between colleague support and thriving among New Zealand ECNs, particularly for Māori and Pacific, underscores the importance of relational work environments for sustaining professional wellbeing and workforce participation. Supportive collegial relationships act as a buffer against stress and emotional exhaustion, enhancing ECNs’ capacity to cope with demanding workloads and organisational pressures. From a workforce sustainability perspective, fostering strong peer networks and culturally responsive support structures may reduce early attrition, particularly among underrepresented groups, and contribute to a more diverse and resilient nursing workforce. Investment in relational and culturally grounded models of support should therefore be viewed as a strategic workforce intervention rather than an optional wellbeing initiative. Initiatives such as structured peer mentoring, team-based models, and embedding principles of whanaungatanga into workplace practices can enhance belonging and wellbeing. Furthermore, investing in leadership development to promote inclusive and supportive team cultures may help mitigate attrition and improve retention among diverse ECNs.

#### 6.1.3. Authenticity at Work

New Zealand ECNs reported higher levels of authenticity at work compared to Australian ECNs; however, authenticity emerged as the only significant predictor of thriving for Australian ECNs. This suggests that while authenticity scored higher among New Zealand ECNs, thriving is influenced by multiple workplace factors, such as colleague support and burnout, while thriving in Australia is primarily centred on the ability to feel authentic in the workplace. Authenticity at work reflects the extent to which individuals can express their true selves, values, and beliefs without fear of negative consequences [54]. As such, in the Australian context, this may indicate that organisational culture and leadership practices play a critical role in enabling ECNs to feel genuine and aligned with their professional identity. In New Zealand, this is not the case. Authenticity at work has important implications for professional wellbeing and long-term career sustainability. These findings suggest that when Australian ECNs are able to align their personal values with their professional roles, they are more likely to experience engagement, meaning, and commitment to the profession. Conversely, environments that constrain authenticity may contribute to moral distress, disengagement, and eventual exit from nursing. Supporting authenticity through inclusive leadership, psychological safety, and respect for professional voice may therefore strengthen ECNs’ sense of professional identity and increase their intention to remain in the workforce over time.

### 6.2. Trans-Tasmin Migration

The results of this study showed little difference in the intention to leave the profession between New Zealand and Australian ECNs, showing optimism that new nurses intend to stay engaged in their newfound vocation. However, levels of intention to leave the organisation would likely be higher in New Zealand ECNs, given that newly qualified nurses are unable to find work since the government implemented significant hiring freezes and cost-cutting measures for healthcare services to address financial challenges [55]. In 2025, only 45 percent of New Zealand mid-year graduates had secured hospital jobs [49]. Further, the lure of higher salaries and better working conditions sees more New Zealand nurses moving across the ditch to Australia each year, with nearly 12,000 making the move in 2024 [55]. First-year nurses in all Australian states and territories receive annual base salaries up to AUD 18,000 higher [47]. The higher cost of living in New Zealand further compounds dissatisfaction with salaries. This is occurring at a time when New Zealand is facing severe nursing shortages. To compound this exodus, 46% of the nursing workforce in New Zealand is made up of internationally qualified nurses due to the immigration fast-track during the COVID-19 pandemic [55]. New Zealand has become a ‘stepping-stone’ country in which these nurses work in New Zealand with the intention of gaining a verification of good standing so they can move on to Australia, enabled by the Trans-Tasman Mutual Recognition Act 1997 (TTMRA) [56].

The ongoing migration of New Zealand nurses to Australia represents a significant challenge to workforce sustainability at a national level. While individual nurses may experience improved wellbeing and job satisfaction through higher pay and safer staffing conditions, the cumulative impact exacerbates shortages within the New Zealand system, increasing workload pressure and burnout for those who remain. This cycle risks further undermining retention and accelerating workforce depletion. Without coordinated policy responses that address pay equity, staffing ratios, and early career support, New Zealand faces continued reliance on internationally qualified nurses and the risk of long-term instability in its domestic nursing workforce.

### 6.3. Recommendations to Improve Organisational Support of Early-Career Nurses

The early results of this study provide insight for the development of innovative theory-informed recommendations to improve organisational support for early-career nurses to increase retention in the profession. First, healthcare organisations should focus on developing policy that helps to reduce burnout in ECNs, particularly in New Zealand. The government needs to increase funding to support the recruitment and retention of the workforce, thereby improving patient ratios and reducing workloads for nurses. Second, organisations should focus on fostering strong collegial connections to promote workplace wellbeing, improve thriving, and reduce intention to leave the profession. In New Zealand, particularly, supporting Māori ECNs should include developing culturally competent workplaces through initiatives that incorporate Māori values and practices into workplace policies. For example, incorporating the concept of whanaungatanga at work brings benefits for Māori and their workplaces because it creates a supportive and inclusive environment [53]. This may include regular informal get-togethers, inviting Māori to participate in decision-making and following a reciprocal ‘give-and-take’ approach when engaging with Māori. Finally, Australian organisations should focus on creating inclusive cultures that allow nurses to express their professional identity without fear of negative consequences. Practical steps include leadership training in authentic management, psychological safety initiatives, and policies that encourage open communication and value diversity. The ‘stepping-stone’ issue that has emerged in New Zealand requires a renewed focus on the barriers and enablers to increase domestic education and recruitment. Further, to curb the loss of nurses to Australia, New Zealand healthcare policy makers need to engage in ongoing examination of remuneration, training capacity, new entry routes and pre-registration attrition [56].

### 6.4. Limitations

This study has several limitations that should be considered when interpreting the findings. The small and unequal sample sizes between the two participating countries limit the generalisability of the results and reduce the ability to make robust cross-country comparisons. Given the small size of the Australian subgroup, the use of multiple regression analysis for this sample should be interpreted with caution; while included to explore potential patterns, the results are preliminary and may not be stable or broadly generalisable. In addition, the voluntary nature of participation introduces the potential for self-selection bias, as individuals with a particular interest or experience related to the study topic may have been more likely to participate. Further, the exclusive reliance on self-reported measures may increase the risk of response bias, including social desirability and recall bias, which could affect the accuracy of the reported data. The findings also come from cross-sectional surveys, limiting assertions about cause–effect relationships. Precautions have been taken to minimise the potential for common-method bias, including measures with well-established construct validity and internal reliability. This study reports early results of the predictors and outcomes of thriving at work; however, research by the international action research programme will take this further and examine what early-career nurses and their managers themselves say should be performed by their organisations to improve their working conditions. Although this study identifies intentions to leave, it does not follow up on actual turnover behaviour, and future research would benefit from such longitudinal results.

## 7. Conclusions

The WHO has emphasised the need for countries to examine how best to achieve national health workforce sustainability, where countries take responsibility to meet their health workforce requirements from their own resources using workforce planning, aligned education and training, and regulation [57]. Overall, the findings of this study reinforce the need for a systems-level approach to nursing workforce sustainability that integrates professional wellbeing, cultural safety, and structural support. Early-career nurses represent a critical investment point for health systems, and failure to address burnout, relational support, and professional identity at this stage may result in preventable loss of these skilled professionals. This study highlights the need for New Zealand to reduce burnout in ECNs by developing a clear planning pathway for improving working conditions that sustain manageable workloads. The challenge lies in nursing ratios, a growing issue as more nurses choose to move ‘across the ditch’ to Australia for better pay and working conditions. Further, organisations in New Zealand should continue to foster workplaces that value the concept of whanaungatanga and social connection.

In Australia, authenticity at work emerged as the strongest predictor of thriving, highlighting the importance of organisational cultures that enable ECNs to express their true selves and professional identity without fear of negative consequences. Strategies that promote psychological safety, inclusive leadership, and authentic communication are essential in supporting ECNs and enhancing retention. Together, these findings highlight the need for tailored approaches in each country to strengthen workforce sustainability and improve ECN wellbeing.

Overall, New Zealand and Australia share many similarities in healthcare standards and workforce challenges; however, important differences in health service structures and workforce policies shape the experiences of ECNs in each country. These differences highlight the need for shared research and cross-country collaboration to fully understand and appreciate how best to address both local and international nursing workforce challenges now and into the future. These coordinated efforts are essential in creating dialogue and developing sustainable strategies that support ECNs while strengthening health systems globally.

## Figures and Tables

**Table 1:** Participant demographics.

Demographic Information	Frequency						
	n	%	n	%	n	%	
	New Zealand		Australia		Total		*p*-Value
**Gender (*n* = 319)**							
- Male	19	7.0%	3	6.3%	22	6.9%	
- Female	201	74.2%	43	89.6%	244	76.5%	0.008 *
- Other	1	0.4%	1	2.1%	2	0.6%	
- Prefer not to say	1	0.4%	1	2.1%	2	0.6%	
- Missing	49	18.1%	0	0.0%	49	15.4%	
**Age (years) (*n* = 325)**							
- 20–29	151	54.5%	20	41.7%	171	52.6%	0.001 **
- 30–39	41	14.8%	13	27.1%	54	16.6%	
- 40–49	24	8.7%	14	29.2%	38	11.7%	
- Missing	61	22.1%	1	2.1%	62	19.1%	
**Ethnicity (*n* = 319)**							
- Aboriginal/Torres Strait Islander	0	0.0%	3	6.3%	3	0.8%	0.001 **
- Asian	68	25.1%	2	4.2%	70	18.5%	
- European/Pākehā	85	31.4%	35	72.9%	120	31.7%	
- South Asian	0	0.0%	2	4.2%	2	0.5%	
- Māori	42	15.5%	2	4.2%	44	11.6%	
- Pacific Peoples	64	23.6%	0	0.0%	64	16.9%	
- MELAR (Middle Eastern/Latin American/African)	7	2.6%	0	0.0%	7	1.8%	
- Other Ethnicity	5	1.8%	4	8.3%	9	2.4%	
**Education level (*n* = 320)**							
- Undergraduate Degree	205	75.4%	45	93.8%	250	78.1%	0.019 *
- Diploma	4	1.5%	0	0.0%	4	1.3%	
- Hospital-Trained	3	1.1%	0	0.0%	3	0.9%	
- Postgraduate Degree	12	4.4%	3	6.3%	15	4.7%	
- Missing	48	17.6%	0	0.0%	48	15.0%	
**Area of work (*n* = 320)**							
- General Medicine	38	14.0%	15	31.3%	53	16.6%	0.001 **
- Intensive Care Unit	20	7.4%	0	0.0%	20	6.3%	
- Emergency Department	16	5.9%	6	12.5%	22	6.9%	
- Child Health	51	18.8%	0	0.0%	51	15.9%	
- Mental Health	27	9.9%	5	10.4%	32	10.0%	
- Surgical	70	25.7%	12	25.0%	82	25.6%	
- Midwife	0	0.0%	4	8.3%	4	1.3%	
- Other	50	18.4%	6	12.5%	56	17.5%	

** *p* ≤ 0.001, * *p* ≤ 0.05.

**Table 2:** Independent sample *t*-test comparing countries.

Predictor Variables	Mean (Standard Deviation)		t	df	*p*-Value
	New Zealand	Australia			
Thriving	4.929 (0.578)	4.902 (0.476)	0.290	311	0.772
- Learning	5.256 (0.578)	5.167 (0.459)	0.966	312	0.335
- Vitality	4.600 (0.808)	4.637 (0.734)	−0.284	311	0.777
Burnout	2.227 (0.734)	1.790 (0.632)	3.643	307	0.001 **
Colleague support	5.622 (0.946)	5.488 (1.104)	0.836	304	0.404
Leadership support	4.897 (1.130)	5.062 (1.068)	−0.878	294	0.381
Organisational support	4.106 (0.886)	4.162 (0.699)	−0.390	294	0.697
Authenticity at work	5.702 (1.938)	5.073 (1.224)	2.001	256	0.046 *
Quality of care	2.671 (1.204)	1.922 (0.538)	3.915	286	0.001 **
Intention to leave profession	4.235 (0.719)	4.235 (0.719)	−1.306	288	0.193

** *p* ≤ 0.001, * *p* ≤ 0.05.

**Table 3:** Factors that impact thriving among New Zealand and Australian early-career nurses.

Factor	R^2^	Predictor	t	AdjOR	95% CI		*p*-Value
New Zealand	0.433	Gender	−2.019	0.925	0.999	1.001	0.167
		Ethnicity	2.350	1.071	0.987	1.061	0.208
		Burnout	−4.219	0.755	0.729	0.891	0.001 **
		Colleague support	5.552	1.448	1.148	1.338	0.001 **
		Organisational support	1.043	1.062	0.968	1.112	0.298
		Authenticity at work	0.895	1.061	0.980	1.054	0.372
Australia	0.797	Gender	1.110	1.145	0.886	1.510	0.275
		Ethnicity	1.090	1.123	0.971	1.100	0.283
		Burnout	−1.842	0.796	0.695	1.017	0.074
		Colleague support	1.594	1.236	0.975	1.232	0.120
		Organisational support	1.772	1.217	0.980	1.338	0.085
		Authenticity at work	2.852	1.480	1.046	1.303	0.007 *

** *p* ≤ 0.001, * *p* ≤ 0.05.

## Data Availability

The data that support the findings of this study are available from the corresponding author, WM, upon reasonable request. The data are not publicly available due to privacy and ethical restrictions.

## Abbreviations

The following abbreviations are used in this manuscript:

ECN	Early-career nurse

## Appendix A

### Survey Instrument

Demographics:
  1. What is your current age (in whole years)?
  2. What is your gender identity?
  3. What is your ethnicity?
  4. What is your highest level of nursing education?
  5. Where did you complete your nursing education?
  6. Please select the option that most closely aligns to your area of work.
  7. How many hours do you usually work each week in your current role?
Thriving at Work Scale:
In relation to your work, rate how you agree with the following statements (7-point scale: 1 = Strongly disagree, 7 = Strongly agree):
  1. I find myself learning often.
  2. I continue to learn more and more as time goes by.
  3. I see myself continually improving.
  4. I am not learning.
  5. I have developed a lot as a person.
  6. I feel alive and vital.
  7. I have energy and spirit.
  8. I do not feel very energetic.
  9. I feel alert and awake.
  10. I am looking forward to each new day.
Maslach Burnout Scale:
When you think of your work overall, how often do you feel the following (5-point scale: 0 = Never, 4 = Always):
  1. Tired
  2. Physically weak/Sickly
  3. Difficulties sleeping
  4. Disappointed with people
  5. Trapped
  6. Worthless/Like a failure
  7. Hopeless
  8. Helpless
  9. Depressed
  10. “I’ve had it”
Colleague Support:
Please tell us about your relationships with colleagues (People you have the most contact with such as registered nurses, healthcare assistants, doctors, and allied health colleagues) (7-point scale: 1 = Strongly disagree, 7 = Strongly agree):
  1. My colleagues appreciate the value of my work and its results.
  2. My colleagues express a positive opinion on my work.
  3. My colleagues give me supportive advice.
  4. My colleagues help me with the performance of my tasks.
Leadership (LMX-7):
We’d like to know about your relationship with your immediate manager (the person who assesses your job performance, e.g., Charge Nurse) (7-point scale: 1 = Strongly disagree, 7 = Strongly agree):
  1. I know where I stand with my manager.
  2. My manager understands my problems and needs.
  3. My manager recognises my potential.
  4. My manager would use their power to help me solve problems at work.
  5. My manager would “bail me out” at their expense.
  6. I have enough confidence in my manager that I would defend and justify their decision if they were not present.
  7. I would characterise my working relationship with my manager as effective
Perceived Organisational Support:
These questions relate to the level of support you receive from your organisation (7-point scale: 1 = Strongly disagree, 7 = Strongly agree):
  1. Senior management really cares about my well-being.
  2. Senior management cares about my general satisfaction at work.
  3. Senior management shows very little concern for me.
Quality of Care:
Regarding your work over the last year, rate your answer to the following statements as honestly as possible. Please remember these answers are anonymous (5-point scale: 1 = Never, 5 = Often):
  1. I make mistakes without negative consequences to patients.
  2. I perform procedures without appropriate training.
  3. I make mistakes with negative consequences to patients.
  4. I fall short in the quality of care I provide to my patients.
  5. I do not have enough time or attention for my patients.
Individual Authenticity at Work:
At work, to what extent can you be who you really are? (7-point scale: 1 = Strongly disagree, 7 = Strongly agree):
  1. In this job, I can express myself.
  2. In this job, I don’t feel I need to hide who I really am.
  3. In this job, I can be myself.
  4. In this job, I don’t have to act like someone I’m not.
  5. In this job, I feel authentic.
  6. In this job, I can be who I really am.
Intention to Leave:
In terms of how you feel now about being a member of the nursing profession, rate your response to the following questions (7-point scale: 1 = Strongly disagree, 7 = Strongly agree):
  1. I want to leave the nursing profession as soon as possible.
  2. If I had it to do over again, I would still go into nursing.
  3. I plan to continue in nursing for the rest of my working life.
